# METTL16 regulates m^6^A methylation on chronic hepatitis B associated gene HLA-DPB1 involved in liver fibrosis

**DOI:** 10.3389/fgene.2022.996245

**Published:** 2022-11-04

**Authors:** Haibing Gao, Xiangmei Wang, Huaxi Ma, Shenglong Lin, Dongqing Zhang, Wenjun Wu, Ziyuan Liao, Mengyun Chen, Qin Li, Minghua Lin, Dongliang Li

**Affiliations:** ^1^ Mengchao Hepatobiliary Hospital of Fujian Medical University, Fujian, China; ^2^ Fuzong Clinical Medical College of Fujian Medical University, Fujian, China; ^3^ 900th Hospital of Joint Logistics Support Forces of the Chinese PLA, Fujian, China

**Keywords:** M6A, METTL16, CHB (chronic hepatitis B), HBV-hepatitis B virus, GWAS

## Abstract

The role of genetic factors in the occurrence and progression of CHB (CHB) is still not fully explored. In recent years, genome-wide association studies on CHB patients have demonstrated that a large number of CHB-associated single nucleotide polymorphisms exist in the gene intron, which may regulate expression at the transcriptional level. Modification of RNA m^6^A methylation is one of the key mechanisms regulating gene expression. Here we show that *METTL16*, an m^6^A regulator involved in mRNA intron splicing, is differentially expressed in CHB the tissue of patients who has definite diagnosis of mild and severe fibrosis. At the same time, there are also significant differences in the expression of CHB-associated genes such as *HLA-DPA1* and *HLA-DPB1*. The expression of *HLA-DPB1* is related to *METTL16*. Furthermore, analyses of RNA binding of METTL16 and *HLA-DPB1* show that the silencing of *METTL16* in astrocytes downregulates m^6^A and expression of HLA-DPB1. In conclusion, *METTL16* participates in the progression of CHB fibrosis by regulating the m^6^A level and expression of HLA-DPB1.

## Introduction

Chronic hepatitis B (CHB) is a chronic inflammatory disease in patients with hepatitis B virus (HBV) infection. The incidence of CHB ranks first among all kinds of infectious diseases (Lok, 2002). More than 1.3 billion people in global are infected with HBV, about 260 million are with CHB, which causes about 1 million deaths every year (Perz et al., 2006; Schweitzer et al., 2015). CHB has become a very serious health and social problem.

Heredity, the virus, and the environment are important factors in the pathogenesis of CHB, which leads to high heterogeneity in clinic. From the perspective of population susceptibility to CHB and disease progression, genetic variation can lead to differences in clinical manifestations among individuals. Since the publication of the first genome-wide association study (GWAS) of CHB in 2009, genetic studies on patients with CHB have revealed many single nucleotide polymorphisms (SNPs) associated with susceptibility to CHB (Raza et al., 2007). Several studies have confirmed that these SNPs are mainly concentrated in a series of human leukocyte antigen (HLA) loci, including *HLA-DP*, *HLA-DQ*, *HLA-C*, and *HLA-DOA* (Lau et al., 2011; Yamada et al., 2014; Akcay et al., 2018). Among them, Mbared et al. found that the SNP rs9277535 with the most significant association with CHB in a Japanese population was located in the 3′ untranslated region of *HLA-DPB1*. The SNP was also identified in Korean, Thai, and Han populations with different significance. Moreover, rs3077, a representative CHB-associated SNP in different populations, is located in the 3′ untranslated region of HLA-DPA1. In addition, SNPs located in *EHMT2*, *TCF19*, *UBE2l3*, *CFB*, *FDX1*, and other gene regions are also associated with susceptibility to CHB in different regions. However, CHB progresses to liver cirrhosis and liver cancer. GWAS shows that variation in the SNP of *HLA* gene closely related to the progression of CHB to liver cirrhosis and participates in the occurrence of liver cancer. Previous studies have shown that the cytotoxicity of HLA class I and class II play an critical role in the spontaneous clearance of HBV. However, the clinical heterogeneity of CHB cannot be fully analyzed from only the level of genetic variation. The associated SNPs vary in different populations, and some findings are difficult to replicate, or even show the opposite results. In the results of GWAS, the genes that play an important role in CHB is not statistically significant. Most SNPs located at *HLA* loci are located in the untranslated region. The functional mechanism is not clear, which may be related to mRNA expression of the gene. SNP loci associated with hepatitis are distributed in the intron region of the gene. From the perspective of SNP–amino acid protein function, the mechanism of action of these SNPs cannot be deeply analyzed. Although it is believed that these SNPs can affect the pathogenesis of CHB by altering gene expression, their key mechanism of action has not been revealed.

Recent studies have found that modification of N6 methyl adenosine (m^6^A) is an important way of controlling gene expression by eukaryotic mRNA. m^6^A modification is mainly distributed in introns and the 3′ untranslated region, especially in region near the stop codon and splice site, which is involved in RNA processing and metabolic function (Liu and Zhang, 2018). m^6^A modification takes part in different stages of development of mRNA (Imam et al., 2018), including RNA folding, stability, splicing, nuclear output, translation regulation, and degradation, to regulate RNA biological function, protein translation, and life activity (Zhao et al., 2021; Tong et al., 2022). m^6^A modification of precursor mRNA mainly takes place in the untranslated region, and m^6^A methylase and reader proteins located in the nucleus. Thus, it can be inferred that m^6^A modification mainly occurs in the nucleus and affects mRNA splicing (Meyer et al., 2012; Zhao et al., 2014; Xu et al., 2017). Knockout of *METTL3* results in the downregulation of introns. In addition, m^6^A demethylase FTO preferentially binds to the RNA intron region, downregulates m^6^A modification on the one hand, but prevents RNA from binding to splicing protein *SRSF2* on the other hand, resulting in abnormal splicing (Dominissini et al., 2012). These studies show that m^6^A modification of RNA in untranslated regions could affects gene expression by regulating RNA processing and metabolism. This phenomenon provides clues for analyzing the role of SNPs in the untranslated region in the pathogenesis of CHB. We speculate that SNPs in the untranslated region impact the occurrence and development of CHB by affecting m^6^A modification and regulating gene expression.

In addition, many studies have shown that m^6^A modification can change expression of important viral genes. Researchers have proven that modification of m^6^A methylation is widely involved in replication of the HBV virus, inflammatory response, immune regulation, and fibrosis and plays a role in liver injury, tumors, and organ failure (Kostyusheva et al., 2021). Imam h et al. mapped the m^6^A site in HBV RNA (Qu et al., 2021; Cheng et al., 2022; Kim et al., 2022; Kim and Siddiqui, 2022; Zhao et al., 2022). m^6^A modification is necessary for efficient reverse transcription of the viral genome and can also regulate the stability of HBV RNA (Kim and Siddiqui, 2021a). Chronic infection with HBV and hepatitis C virus is the main cause of hepatocellular carcinoma (Xiao et al., 2016; Xu et al., 2017). There is increasing evidence that hepatocellular carcinoma oncoproteins induced by both virus are controlled by m^6^A modification. Recent works found that m^6^A modification involves the regulation of hepatocellular carcinoma through METTL3 and METTL14. First, Chen et al. (2018) observed the expression of *METTL3* increased abnormally in liver cancer and increased cell proliferation *in vitro*, resulting in promoted tumorigenicity *in vivo* (Xu et al., 2017). METTL3 is significantly upregulated in hepatocellular carcinoma and promotes tumor progression. It inhibits SOCS2 expression and promotes cancer cell proliferation and metastasis through the m^6^A-YTHDF2 mechanism. Chen et al. (2018) found interference with METTL3 reduce the expression of SOCS2 mRNA. Second, it was reported that METTL14 is downregulated in liver cancer, and thereby regulates the development of liver cancer (Bartosovic et al., 2017; Ma et al., 2017). Together these evidences suggest that m^6^A modification has a key role in liver-related diseases through various m^6^A-related proteins (Wu et al., 2019; Wu et al., 2020; Kim and Siddiqui, 2021b; Wang and Zhou, 2022). Modification of m^6^A methylation is involved in the pathogenesis of liver injury, organ failure, and fibrosis. However, it is unclear whether it is involved in the development of CHB.

Here, we investigated the expression of m^6^A regulator in different stages of CHB, examined the relationship between m^6^A and CHB-associated genes, and checked the change in m^6^A and expression of gene loci with CHB-associated SNPs.

## Materials and methods

### Patients

The ethical approval was approved by the ethics committee of Mengchao Hepatobiliary Hospital of Fujian Medical University and all study participants obtained informed consent. Clinical data were collected from patients with CHB diagnosed by liver biopsy in our hospital in 2019 or 2020. The diagnostic criteria were in accordance with the guidelines for the prevention and treatment of CHB (2019 Edition), and study subjects provided informed consent before enrollment. Inclusion criteria were 1) being HBsAg positive for more than 6 months and HBsAb negative and 2) being between 18 and 60 years old. Exclusion criteria were 1) the presence of acute hepatitis B, liver failure, or primary liver cancer, in combination with drug liver, alcoholic liver, or fatty liver, in combination with any other viral infection and other serious disease; 2) use of antiviral drugs up to 3 months before enrollment; 3) receipt of immunosuppressant and immunomodulator treatment up to 6 months before enrollment; 4) autoimmune liver disease and systemic autoimmune disease; and 5) pregnancy.

### Specimens

A BARD puncture biopsy gun (with a sampling length of 2.2 cm) and 16 g disposable cutting biopsy needle were used for the liver puncture biopsy. One tissue specimen was stained with he, Masson, and reticular fibers, and a single pathologist read the film uniformly according to the pathological diagnostic criteria. The other specimen was kept in the refrigerator at −80°C.

### Tandem mass spectrometry (LC/MS)

After total RNA is extracted with Trizol, mRNA can be enriched with Oligo (dT) magnetic beads. RNA was digested from a single strand into a single base with nuclease P1. Alkaline phosphatase and ammonium bicarbonate were added, the sample was allowed to incubate for several hours, and then the sample was injected into a liquid chromatograph. Finally, the overall degree of m^6^A methylation on mRNA was calculated according to the ratio of m^6^A to total adenine.

### Real-time fluorescence quantitative PCR

Tissues or cells were digested and lysed by Trizol reagent. After Trizol was added to cells or tissues, total RNA was extracted with chloroform isopropanol extraction. cDNA was synthesized by reverse transcription with a one-step PrimeScript cDNA synthesis kit. Quantitative PCR was performed with a one-step SYBR PrimeScript RT-PCR kit. GAPDH was used as the internal reference gene, and the quantitative results were 2^−ΔΔCT^ indicates. The primer information was in (Supplemental Table S1).

### meIP-PCR

The combination of immunoprecipitation (ChIP) and PCR technology can be utilized to efficiently determine the interaction *in vivo*. RNA was isolated and broken into small fragments by ultrasounication. An specific antibody was added, and the antibody formed an immune binding complex with the target protein. De crosslinking, RNA purification and qPCR were further processed.

### Statistical analysis

SPSS 20.0 was used for statistical the analysis. The measurement data conforming to normal distribution adopts mean ± standard deviation (±s). *t* tests were used for pairwise comparisons of normally distributed data. Single-factor analysis of variance was used for multigroup comparisons. Spearman correlation analysis was used to analyze correlations between various factors and the occurrence and degree of liver fibrosis in patients with CHB.

## Results

### SNPs associated with susceptibility to CHB are located in different genes

**FIGURE 1:**
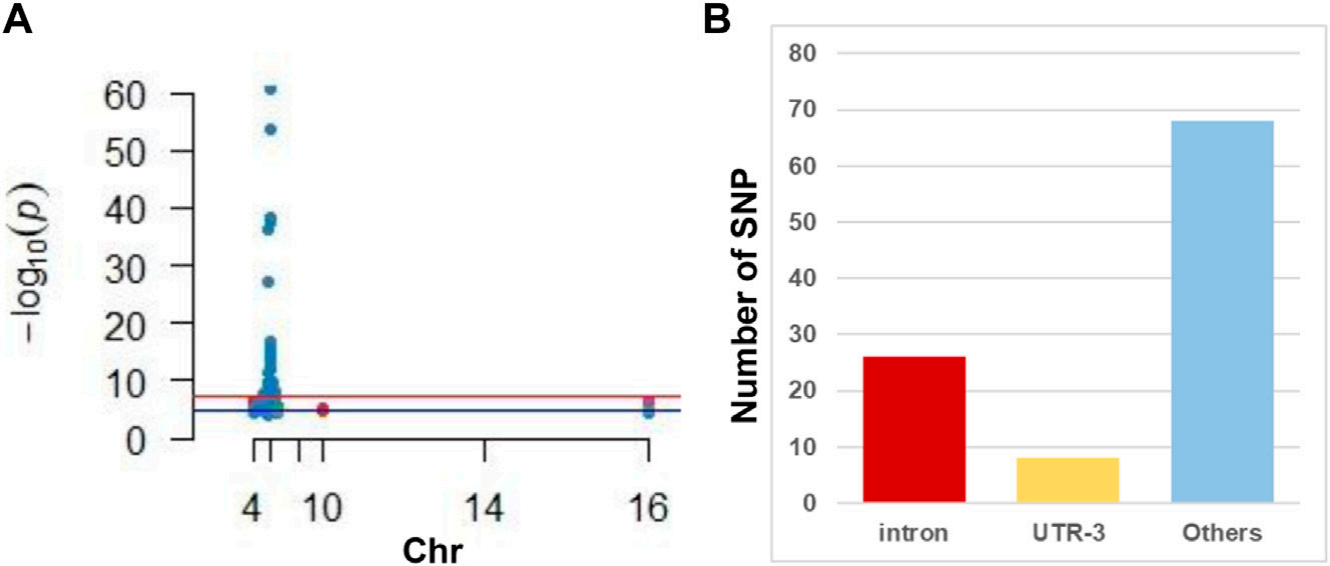
Illustration of GWAS studies in CHB. **(A)** Manhattan plot of CHB associated SNP reviewed from published literature. Note that most SNPs located in the chromosome 6. **(B)** Distribution of SNP in gene different regions. Note that intron is hot regions where CHB associated SNP frequently located.

GWAS has identified 102 SNP sites related to susceptibility to or progression of CHB (Figure 1A and Supplemental Table S2). We discovered that only three SNPs were distributed in the exon region of the gene, nearly 26 were distributed in the intron region of the gene, and the rest were distributed in the 3′ and 5′ untranslated regions (Figure 1B).

### Patients show different levels of liver fibrosis

**FIGURE 2:**
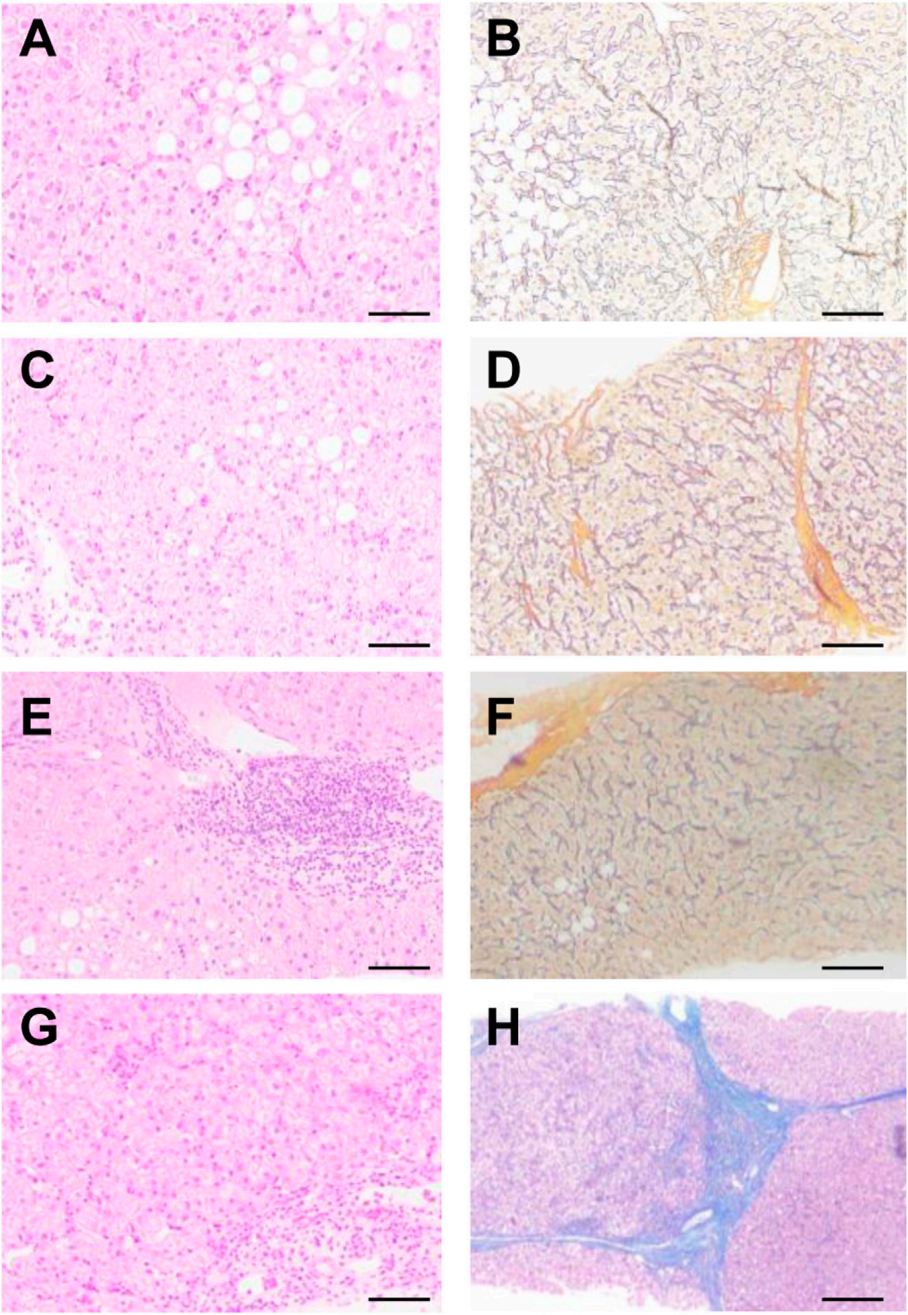
Pathological analysis of patients with different levels of liver fibrosis. According to Ishak scoring **(A,B)** s1, **(C,D)** s2, **(E,F)** s4, **(G,H)** s5. **(A,C,E,G)** HE, **(B,D,F,H)** Masson.

A BARD puncture biopsy gun (with a sampling length of 2.2 cm) and 16 g disposable cutting biopsy needle were used for the liver puncture biopsy. Two complete liver tissues with a length of about 1.5–2.0 cm were taken. One tissue sample was sectioned consecutively into five pieces; and stained with conventional HE staining, Masson staining, and reticular fibers. A single pathologist read the film uniformly according to the pathological diagnostic criteria and divided the films into a mild fibrosis group (s1–s2) and a severe fibrosis group (s4–s5) according to Ishak scoring criteria (Figure 2).

### METTL16 is differentially expressed in the mild and severe fibrosis groups

**FIGURE 3:**
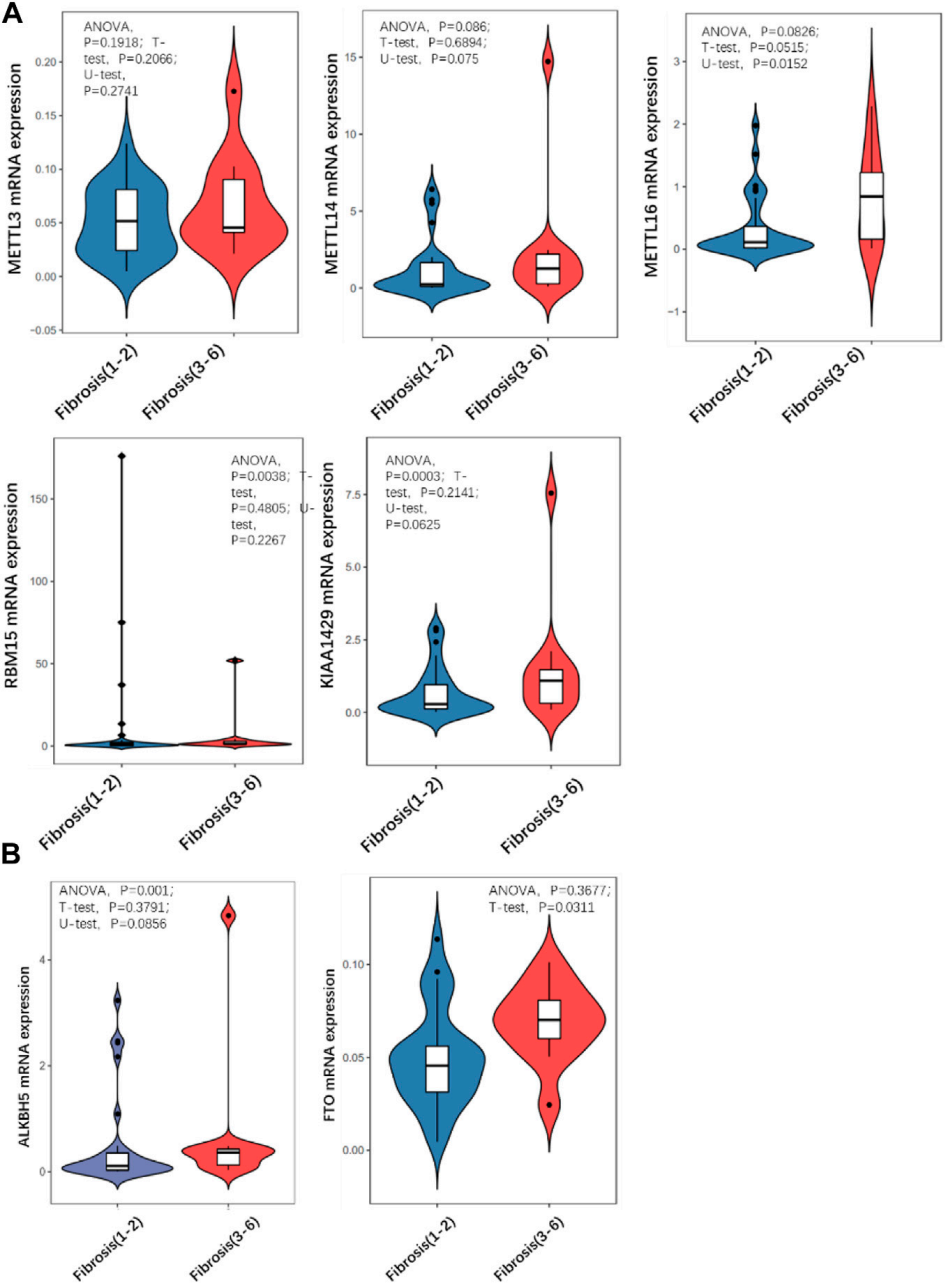
Comparison of expression level of m^6^A regulator in mild and severe fibrosis groups. **(A)** writers, **(B)** erasers. Note that METTL16 is significantly up-regulated in severe fibrosis group.

Quantitative PCR was carried out to detect the expression level of a series of m^6^A methyltransferase regulator genes. *METTL16* expression was significantly higher in the severe group than in the mild group (Figure 3A). The expression of other m^6^A demethyl regulators were also checked, and there was no statistically significant differences. Then we detected the m^6^A modification level of total RNA in the two groups by LC/MS and found that it was significantly (more than 2 times) higher in the severe group than in the mild group (Figure 3B).

### HLA-DPB1 is differentially expressed in fibrosis groups

**FIGURE 4:**
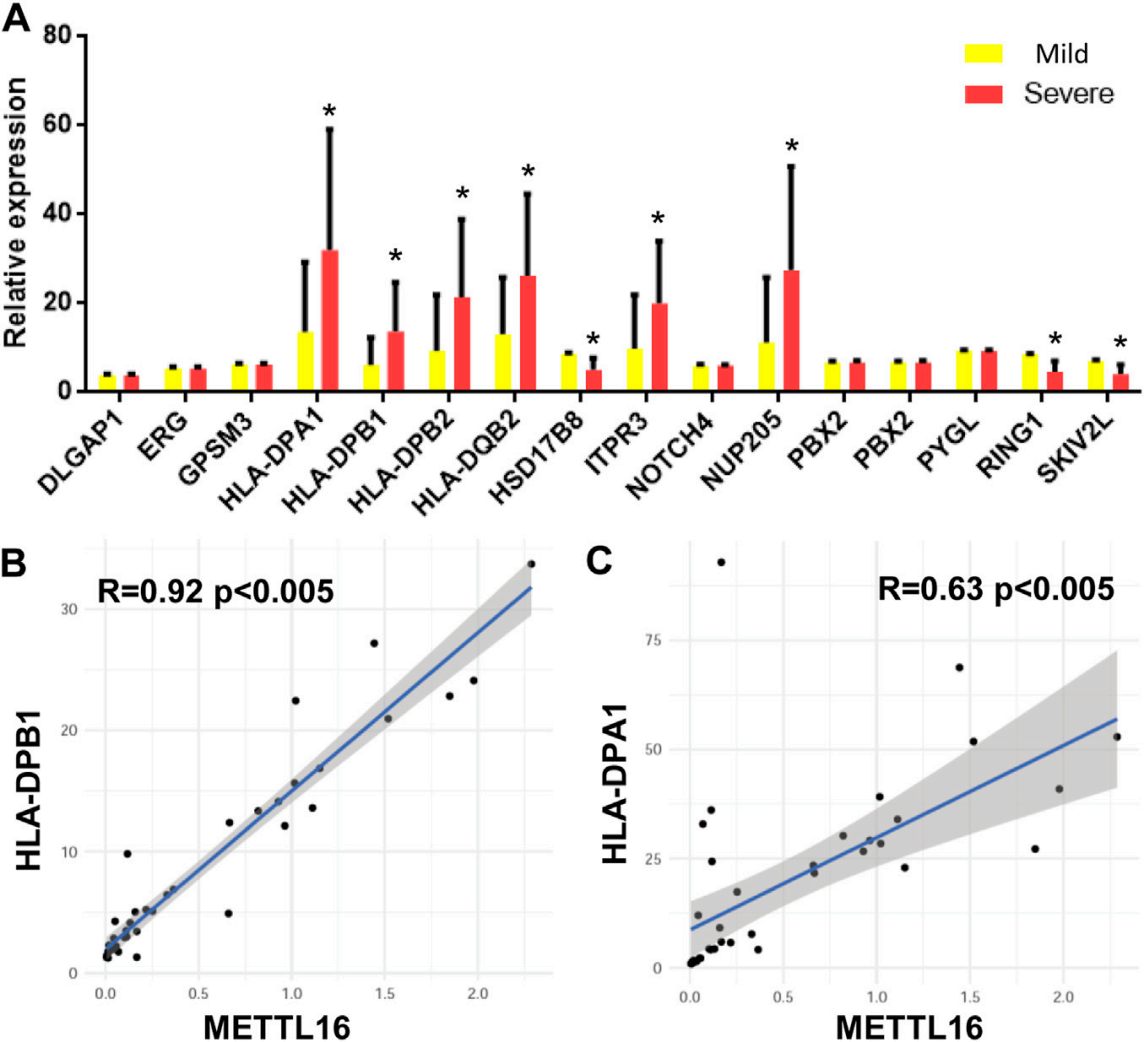
CHB GWAS genes differentially expressed between mild and severe fibrosis groups. **(A)** expression level of CHB GWAS genes. Note that HLA-DPB1 is up-regulated in severe fibrosis group. **(B,C)** METTL16 is co-expressed with HLA-DPB1 and HLA-DPA1.

As mentioned earlier, SNPs related to CHB are located in different genes in the genome according to GWAS. The expression of 15 genes was detected in each sample by quantitative RT-PCR. A total of eight genes were significantly differentially expressed in the two groups of samples. That is, HLA-DPA1, HLA-DPB1, HLA-DPB2, HLA-DQB2, ITPR3, and NUP205 were upregulated in the severe group. In contrast, HSD17B8, RING1, and SKIV2L were downregulated in the severe group (Figure 4A).

The relationships between these differentially expressed genes and the expression of m^6^A regulators were analyzed by Pearson correlation analysis. mettl16 was significantly positively correlated with HLA-DPB1 and HLA-DPA1 (Figures 4B,C).

### There are different levels of m^6^A on HLA-DPB1 in the mild and severe fibrosis groups

**FIGURE 5:**
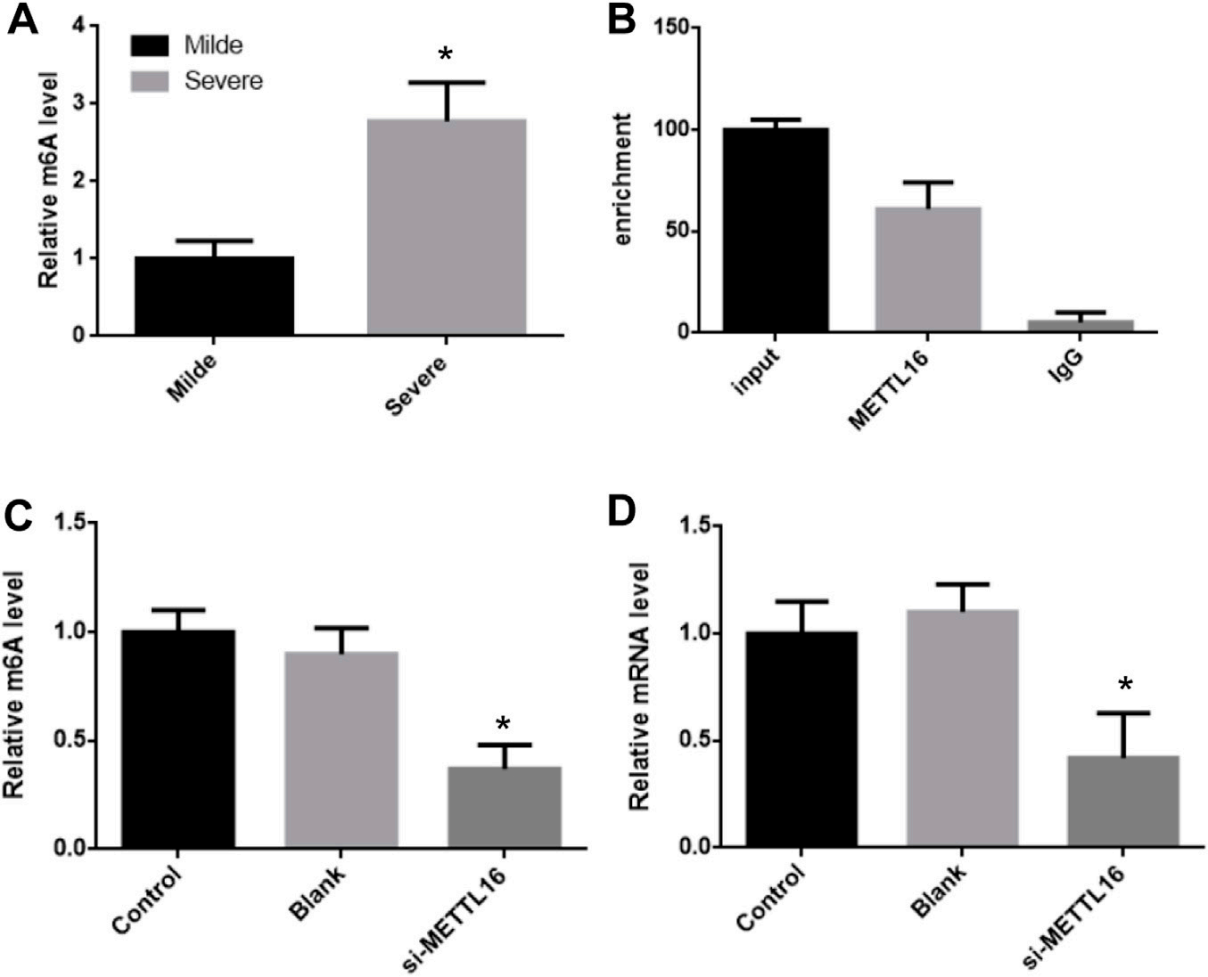
METTL16 control HLA-DPB1 expression through regulating m^6^A level. **(A)** m^6^A level of HLA-DPB1 mRNA between mild and severe fibrosis groups. **(B)** METTL16 interact with HLA-DPB1 mRNA. **(C,D)** knock-down METTL16 reduced m^6^A and mRNA of HLA-DPB1 in hepatic stellate cells.

It was suggesting that the expression of HLA-DPB1 is related to the level of RNA m^6^A. The m^6^A level of HLA-DPB1 in each sample was detected by MeIP qPCR. The level of m^6^A on HLA-DPB1 mRNA was significantly increased in the severe group than in the mild group (Figure 5A).

### METTL16 interacts with HLA-DPB1 mRNA

The m^6^A level of *HLA-DPB1* mRNA was consistent with its expression in each group and was also related to the expression of mettl16. This implies that mettl16 may be one of the causes of the difference in m^6^A level and expression of *HLA-DPB1*. First RNAip experiments showed that mettl16 could bind to HLA-DPB1 mRNA (Figure 5B).

Then we silenced the expression of METTL16 in hepatic stellate cells and detected the expression of HLA-DPB1 and the degree of m^6^A modification. In the METTL16 silencing group, the m^6^A level of HLA-DPB1 mRNA was significantly downregulated by more than 2 times (Figures 5C,D).

## Discussion

Molecular genetics research on CHB has revealed a large amount of genetic information that is of great value for obtaining a complete understanding of the pathogenesis of CHB and the development of innovative treatments. Especially in the past 2 decades of population genetics research, a large number of SNPs related to susceptibility to and progression of CHB have been found through GWAS. Most of these studies have been conducted in Asian populations, and their conclusions are well targeted. The high prevalence of CHB in Asia can be further understood from these research results. The SNPs found in these GWASs are mainly concentrated in HLA loci, including HLA-DPA1, -DPB1, -DQB2, and -DPB2. As an important gene group that regulates the body’s immune response, the HLA complex participates in the anti-HBV immune response, affects the chronicity of HBV infection and the strength of the immune response, and participates in the progression of CHB to cirrhosis and liver cancer. Therefore, the expression of these genes is likely closely related to CHB. In our study, we found that HLA-DPA1 and HLA-DPB1 differed significantly in groups with different degrees of liver fibrosis. This result suggests that the expression of these two genes may be involved in mediating the progression of CHB. In addition, we found that other CHB-related loci, such as HSD17b8, ITPR3, NUP205, RING1, and SKIV2l, were upregulated or downregulated in different ways in the groups with different degrees of liver fibrosis. This shows that controlling the expression of CHB-related genes at the transcriptional level is of great significance for regulating the progression of CHB. However, we found a large number of CHB-associated SNPs found in GWAS were located in the noncoding region of the locus, which suggests that these genes may be involved in regulating CHB at the transcriptional level rather than the function of the encoded protein. In conclusion, our data show that genes with CHB-associated SNPs can participate in the mechanism of CHB through transcriptional regulation.

m^6^A modification plays an vital role in transcriptional regulation in eukaryotes. The stability, transportation, splicing, and translation efficiency of mRNA are closely related to the degree of m^6^A modification. This modification is regulated by the complex. METTL3, METTL14, WTAP, and KIAA1429 form the “writer,” whereas alkbh4 and FTO form the “eraser.” These usually regulate the modification of mRNA in the coding region and the 3′ or 5′ end. Recent studies have found that RNA has m^6^A modifications in the intron region, which affects the splicing of mRNA. Mettl16 is a key methyltransferase whose precursor mrnam^6^a modification affects intron cleavage. In our study, key regulatory factors of m^6^A, especially mettl16, were differentially expressed in tissues with different degrees of liver fibrosis, although other m^6^A regulators did not differ significantly. This shows that m^6^A participates in the regulation of CHB mainly through mett16. However, the GWASs summarized above found that SNPs associated with CHB are mainly located in the noncoding region of the gene. This is consistent with the function of mettl16. We further found that mettl16 could bind to HLA-DPB1 mRNA and change its m^6^A modification level and expression. In clinical samples, the expression of METTL16 was also correlated with HLA-DPB1. All these findings suggest that mettl16 may affect CHB by regulating the expression of these CHB-associated loci, a new mechanism in the process of CHB that needs to be analyzed further.

## Data Availability

The original contributions presented in the study are included in the article/Supplementary Material, further inquiries can be directed to the corresponding authors.
